# Safety of Tumor Treating Fields (TTFields) therapy in pediatric patients with malignant brain tumors: Post-marketing surveillance data

**DOI:** 10.3389/fonc.2022.958637

**Published:** 2022-07-29

**Authors:** Stewart Goldman, Ashley Margol, Eugene I. Hwang, Kazuhiro Tanaka, Bogdana Suchorska, John R. Crawford, Santosh Kesari

**Affiliations:** ^1^ Phoenix Children’s Hospital, University of Arizona College of Medicine, Phoenix, AZ, United States; ^2^ Children’s Hospital Los Angeles, Keck School of Medicine of University of Southern California, Los Angeles, CA, United States; ^3^ Department of Oncology, Children’s National Hospital, Washington, DC, United States; ^4^ Department of Neurosurgery, Kobe University, Kobe, Japan; ^5^ Department of Neurosurgery, Sana Kliniken Duisburg, Duisburg, Germany; ^6^ Department of Neurology, Children’s Health of Orange County, Orange County, CA, United States; ^7^ Providence Southern California Research Clinical Institute, Saint John’s Cancer Institute, Pacific Neuroscience Institute, Los Angeles, CA, United States

**Keywords:** pediatric, brain tumor, Tumor Treating Fields, glioblastoma, safety, adolescents

## Abstract

There is an unmet need to develop effective and tolerable treatments for pediatric patients with malignant central nervous system tumors. This is especially essential for pediatric patients with aggressive brain tumors such as high-grade gliomas, which have a typical survival rate of under 2 years. Tumor Treating Fields (TTFields) are locoregional, noninvasive electric fields that produce an antimitotic effect on cancerous cells when applied to the skin *via* arrays. TTFields therapy (200 kHz) is currently approved in adult patients with newly diagnosed glioblastoma (GBM), with temozolomide, and recurrent GBM as monotherapy. Positive preclinical and clinical data have encouraged off-label use of TTFields therapy in pediatric patients with brain tumors, and this study aims to explore the safety of TTFields therapy in pediatric patients (0–18 years of age) based on data from an unsolicited post-marketing surveillance safety database. The real-world data reported here demonstrate that TTFields therapy has a favorable safety profile for pediatric patients with brain tumors, with no new safety signals observed. Findings from this study warrant further research into the efficacy of TTFields therapy, as well as its potential impact on the quality of life in pediatric patients.

## Introduction

Malignant brain tumors are the most common solid cancer in children and adolescents globally (1). The prognosis for high-grade gliomas (HGGs) is particularly poor, with a median overall survival (OS) of around 2 years (2–6).

Although there have been some advances in the treatment of pediatric central nervous system (CNS) tumors, there are limited options for aggressive tumors such as HGGs (3). Currently, there is no standard of care for this patient population (<18 years of age) beyond resection and focal irradiation (7), with the rarity and heterogeneity of these tumors acting as barriers to the development of new therapies (8). Additionally, molecular transport of medication across the blood-brain barrier (BBB) is highly restricted, thus delivering drugs into the CNS is difficult (9). Since pediatric and adult brain tumors often share the same general pathology, adult treatment strategies – such as biopsy, partial resection, or total resection followed by radiation therapy with or without temozolomide (TMZ) – have traditionally been applied to pediatric patients (10). However, it is now recognized that glioblastomas (GBM) occurring in pediatric and adult patients are genetically distinct: *EGFR*, *TERT*, and *PTEN* mutations are often seen in adult patients, whereas *NTRK*, *H3K27M*, *H3G34R*, and *H3G34V* mutations are commonplace in pediatric patients (11). Thus, there is an unmet need for effective and tolerable treatments directed towards pediatric patients with HGGs and other aggressive CNS cancers.

**Figure 1:**
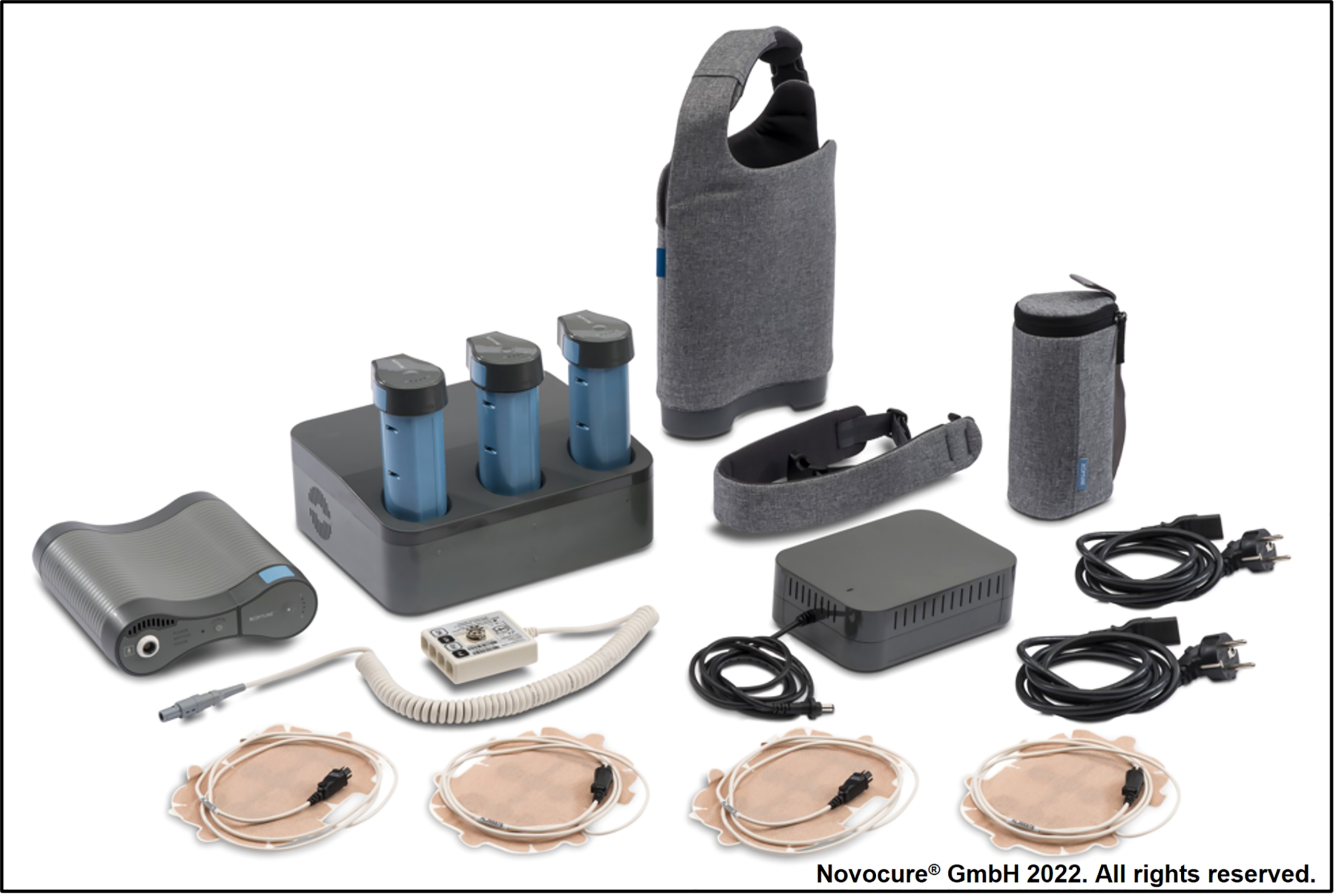
The portable NovoTTF-200A System delivers TTFields using four skin-placed arrays.

Tumor Treating Fields (TTFields) therapy is a locoregional, noninvasive anti-cancer treatment modality approved for the treatment of newly diagnosed (nd) and recurrent (r) GBM, as well as unresectable malignant pleural mesothelioma in adult patients (12–16). TTFields are electric fields that exert antimitotic effects on cancerous cells, produced by a portable, battery-powered generator and delivered to the tumor by skin-placed arrays positioned close to target the tumor site. A recent upgrade of the device was made available for the treatment of GBM; the second-generation NovoTTF-200A System is smaller and lighter weight than the first-generation NovoTTF-100A System (2.7 lbs vs. 6 lbs) (**Figure 1**) (17).

TTFields therapy approval in GBM was based on the results of the EF-11 and EF-14 phase 3 pivotal studies, which evaluated the safety and efficacy of TTFields monotherapy in rGBM, and TTFields therapy concomitant with maintenance TMZ in ndGBM, respectively (18, 19).

EF-11 was a prospective, randomized controlled clinical study evaluating the efficacy and safety of TTFields monotherapy versus physician’s choice of best standard of care (BSC) in adult patients with rGBM. TTFields monotherapy showed clinical benefit comparable to chemotherapy in patients with rGBM. Median survival with TTFields therapy versus BSC was 6.6 versus 6.0 months (hazard ratio [HR], 0.86 [95%] confidence interval (CI): 0.66–1.12]; *p* = 0.27), 1-year survival rate was 20% versus 20%, and progression-free survival (PFS) rates at 6 months were 21.4% and 15.1% (*p* = 0.13), respectively. TTFields therapy-related adverse events (AEs) included localized mild and moderate skin rash beneath the arrays. Patients in the group treated with TTFields therapy experienced fewer treatment-related AEs and systemic AEs than those in the BSC group. Quality of life (QoL) favored the use of TTFields therapy over BSC in the cognitive and emotional function domains, with no significant differences between TTFields therapy and BSC in the global health and social functioning domains. Furthermore, symptom scale analysis regarding treatment-associated AEs – appetite loss, diarrhea, constipation, nausea, and vomiting – were directly related to chemotherapy administration. Importantly, increased pain and fatigue was only reported by chemotherapy-treated patients (18).

EF-14 was a prospective, randomized, controlled clinical study comparing TTFields therapy plus maintenance TMZ to TMZ alone in adult patients with resected or biopsied ndGBM who had completed chemoradiotherapy with concomitant TMZ. Results revealed a statistically significant improvement in PFS (6.7 vs. 4.0 months; HR 0.63 [95% CI: 0.52–0.76]; *p* < 0.001) and OS (20.9 vs. 16.0 months; HR 0.63 [95% CI: 0.53–0.76]; *p* < 0.001) with TTFields therapy plus maintenance TMZ versus TMZ alone. These improved outcomes were obtained without significant increase in the frequency of systemic AEs with the addition of TTFields therapy (19). Consistent with QoL results from EF-11, TTFields therapy in this study did not have a negative impact on QoL, with the exception of skin being more itchy underneath the arrays, which is expected and consistent with the known safety profile of TTFields therapy (20).

Although TTFields therapy is approved for the treatment of adult GBM, the label does not include use in pediatric patients, since the populations of the pivotal EF-11 and EF-14 trials ≥18 years of age (12, 14, 16, 21). Promising preclinical data demonstrating the efficacy of TTFields therapy in various pediatric brain tumor cell lines, including GBM, medulloblastoma, and ependymoma (22), together with strong clinical efficacy in adult patients with GBM (18, 19), have resulted in some off-label use of TTFields therapy in pediatric patients. Resulting data show that, as with adults, TTFields therapy-related AEs in children were dermatologic in nature and mostly mild or moderate manageable events (23–26). Retrospective post-marketing surveillance data from patients who received TTFields therapy, which included a pediatric population aged <18 years (N = 52), revealed no new safety concerns and a safety profile comparable to clinical trials; AE incidence was lower in pediatric patients versus adult and elderly patients (23). Furthermore, preliminary results from the Pediatric Brain Tumor Consortium 048 (PBTC-048) study also showed good tolerability in 11 pediatric patients with recurrent supratentorial HGG and ependymoma. Due to the success of the study, a protocol amendment will allow the use of an alternative array arrangement to target diffuse intrinsic pontine glioma (DIPG) (27).

Taken together, available data in pediatric cell lines and pediatric patients provide a rationale for investigating the feasibility and safety of TTFields therapy in this patient population. Here, we report post-marketing surveillance safety data from pediatric patients with brain tumors, treated with TTFields therapy in the real-world clinical setting.

## Methods

### Data collection

Unsolicited post-marketing surveillance safety data from pediatric patients with brain tumors, treated with TTFields therapy, were obtained from the device manufacturer’s (Novocure^®^) safety database, and retrospectively analyzed. Data from patients <18 years of age with brain tumors who received TTFields therapy between January 1, 2015 to August 31, 2021 in Europe, the Middle East and Africa, Japan, and the USA, were included. As of 2016, the World Health Organization (WHO) deemed the term ‘pediatric GBM’ obsolete due to the wide underlying molecular and genetic diversity in GBM; in this manuscript, the term GBM is used to represent the diagnosis from the patients’ physicians (28).

AE data were obtained during interactions between patients, caregivers, and healthcare professionals and the device manufacturer, as well as, Device Support Specialist visits, prescriber interactions, and patient emails to the nCompass™ support team.

### Data analysis

Safety reports were assessed by Novocure’s Medical Safety department according to the health authorities’ regulations. AEs were classified by system organ class and preferred term using the Medical Dictionary for Regulatory Activities (MedDRA) version 24.0. AEs were also evaluated for severity and relatedness to TTFields therapy. AE relatedness was assessed by Novocure’s Medical Safety team based on the number of unique patients reporting an AE. Due to the retrospective study design, AEs could only be classified as serious or nonserious. An AE was considered serious if it met ≥1 of the following criteria: (1) death; (2) life-threatening pathology; (3) persistent or significant disability or incapacity; (4) in-patient hospitalization or prolongation of existing hospitalization; (5) congenital abnormality or birth defect; or (6) medical/surgical intervention to prevent life-threatening illness, injury, or permanent body structure/function impairment. Safety data were also screened for new safety signals.

### Presentation of results

Data were analyzed for the whole population by age: children (<13 years), adolescents (13–17 years), and by diagnosis: newly diagnosed or recurrent. Data were presented as the number of events and the number of unique patients reporting an event (incidence). Due to the retrospective nature of the analysis, statistical testing was descriptive.

## Results

Overall, 81 patients were included in the analysis. Patient numbers and baseline characteristics were balanced across age groups (**Table 1**). The proportions of patients with newly diagnosed versus recurrent brain tumors were similar (51% vs. 47%) and the majority of patients had supratentorial (vs. infratentorial) tumors (88% vs. 12%). A substantial proportion of patients had tumors classified as HGGs, including anaplastic astrocytoma, anaplastic ependymoma, and GBM (**Table 1**).

**Table 1:** Baseline characteristics of pediatric patients with malignant brain tumors (N = 81).

Characteristic*	Age subgroups		Total (N = 81)
	Children (<13 years of age)(n = 40)	Adolescents (13–17 years of age)(n = 41)	
a Median Age, years (range)	10 (3–12)	15 (13–17)	13 (3–17)
Sex, n (%)			
Male	29 (73)	25 (61)	54 (67)
Female	11 (28)	16 (39)	27 (33)
Region, n (%)			
United States	28 (70)	31 (76)	59 (73)
EMEA^†^	12 (30)	9 (22)	21 (26)
Japan	0	1 (2)	1 (1)
Newly diagnosed, n (%)	18 (45)	23 (56)	41 (51)
Anaplastic astrocytoma^‡^	6 (15)	2 (5)	8 (10)
Glioblastoma^‡^	12 (30)	20 (49)	32 (40)
High-grade glioma (not otherwise specified)^‡^	0	1 (2)	1 (1)
Recurrent disease, n (%)	21 (53)	17 (42)	38 (47)
Anaplastic astrocytoma^‡^	3 (8)	2 (5)	5 (6)
Anaplastic ependymoma^‡^	1 (3)	1 (2)	2 (3)
Atypical meningioma	1 (3)	0	1 (1)
Glioblastoma^‡^	13 (33)	14 (34)	27 (33)
HGG (not otherwise specified)^‡^	2 (5)	0	2 (3)
PNET	1 (3)	0	1 (1)
Unknown diagnosis, n (%)	1 (3)	1 (2)	2 (3)
HGG (not otherwise specified)^‡^	1 (3)	0	1 (1)
Pleomorphic xantoastrocytoma	0	1 (2)	1 (1)
Tumor location, n (%)			
Supratentorial	35 (88)	36 (88)	71 (88)
Infratentorial	3 (8)	1 (2)	4 (5)
Unknown	2 (5)	4 (10)	6 (7)

*Where percentages are provided, they are for the total in the subgroup throughout (children or adolescents) and rounded to the nearest integer so may not equate to 100%.

^†^Austria, Germany, Israel, Italy, and Switzerland.

^‡^Considered HGGs.

EMEA, Europe, Middle East, and Africa; GBM, glioblastoma; HGG, high-grade glioma; PNET, primitive neuroectodermal tumor.

In total, 51 (63%) patients experienced ≥1 AE, with 170 events recorded. Frequency and types of AEs were similar across age groups. Skin reactions (36%) were the most common AE overall and were similar within each age group (children: 35%, adolescents: 37%), but slightly higher in patients with newly diagnosed versus recurrent disease (68% vs. 58%, respectively) (**Tables 2**, **3**). Twenty-eight serious AEs (SAEs) occurred in 11 (14%) patients in the total cohort: seven (18%) in the children cohort and four (10%) in the adolescent cohort. In the total cohort, seizure (5%), infection (4%), and brain edema (2%) were the most frequently observed SAEs (**Tables 4**, **5**). Within the adolescent cohort, there were no cases of ≥ 1 patient reporting a SAE. No SAE was deemed to be related to TTFields therapy. Thirty-seven deaths were reported as of the data cut-off point, none of which were related to device use.

**Table 2:** AEs occurring in ≥5% of pediatric patients with malignant brain tumors, by age.

Preferred term, n (%)	Total events (n = 170)	Age subgroups		Total(N = 81)
		Children (<13 years of age) (n = 40)	Adolescents (13–17 years of age) (n = 41)	
Patients with ≥1 AE	–	23 (58)	28 (68)	51 (63)
Skin reaction	48	14 (35)	15 (37)	29 (36)
Electric sensation*	14	3 (8)	5 (12)	8 (10)
Headache	12	2 (5)	9 (22)	11 (14)
Heat sensation^†^	11	3 (8)	7 (17)	10 (12)
Seizure	10	5 (13)	3 (7)	8 (10)
Nausea/vomiting	6	0 (0)	5 (12)	5 (6)
Fatigue/malaise	5	3 (8)	2 (5)	5 (6)

Brain tumors included anaplastic astrocytoma, anaplastic ependymoma glioblastoma, atypical meningioma, high-grade glioma (not otherwise specified), and pleomorphic xantoastrocytoma.

*Beneath array tingling sensation; tingling.

^†^Beneath array warm sensation; warmth.

AE, adverse event.

**Table 3:** AEs occurring in ≥ 5% of pediatric patients with malignant brain tumors, by diagnosis.

Preferred term, n (%)	Total events (n = 170)	Diagnosis		Total(N = 81)
		Newly diagnosed (n = 41)	Recurrent (n = 40)	
Patients with ≥1 AE	–	28 (68)	23 (58)	51 (63)
Skin reaction	48	19 (46)	10 (25)	29 (36)
Electric sensation*	14	4 (10)	4 (10)	8 (10)
Headache	12	6 (15)	5 (13)	11 (14)
Heat sensation^†^	11	6 (15)	4 (10)	10 (12)
Seizure	10	4 (10)	4 (10)	8 (10)
Nausea/vomiting	6	3 (7)	2 (5)	5 (6)
Fatigue/malaise	5	3 (7)	2 (5)	5 (6)
Fall	3	3 (7)	0	3 (4)
Infection	3	1 (2)	2 (5)	3 (4)
Alopecia	2	2 (5)	0	2 (2)
Balance disorder	2	2 (5)	0	2 (2)
Hemiparesis	2	0	2 (5)	2 (2)
Hypersomnia	2	0	2 (5)	2 (2)
Hypertension	3	1 (2)	2 (5)	3 (4)
Tachycardia	2	0	2 (5)	2 (2)

Brain tumors included anaplastic astrocytoma, anaplastic ependymoma glioblastoma, atypical meningioma, high-grade glioma (not otherwise specified), and pleomorphic xantoastrocytoma.

*Beneath array tingling sensation; tingling.

^†^Beneath array warm sensation; warmth.

AE, adverse event.

**Table 4:** SAEs in pediatric patients with malignant brain tumors, by age.

Preferred term, n (%)	Total events (n = 28)	Age subgroups		Total(N = 81)
		Children (<13 years) (n = 40)	Adolescents (13–17 years) (n = 41)	
Patients with ≥1 SAE	–	7 (18)	4 (10)	11 (14)
Seizure	5	5 (13)	0	5 (6)
Infection	3	2 (5)	1 (2)	3 (4)
Brain edema	2	2 (5)	0	2 (2)
Hypertension	2	1 (3)	1 (2)	2 (2)
Tachycardia	2	1 (3)	1 (2)	2 (2)
Adverse drug reaction	1	0	1 (2)	1 (1)
Colitis	1	1 (3)	0	1 (1)
Nausea/vomiting	1	0	1 (2)	1 (1)
Respiratory tract infection	1	1 (3)	0	1 (1)
Altered state of consciousness	1	1 (3)	0	1 (1)
Cerebral hemorrhage	1	0	1 (2)	1 (1)
Headache	1	0	1 (2)	1 (1)
Hemiparesis	1	0	1 (2)	1 (1)
Hypersomnia	1	0	1 (2)	1 (1)
Paresis	1	1 (3)	0	1 (1)
Speech disorder	1	1 (3)	0	1 (1)
Dyspnea	1	0	1 (2)	1 (1)
Hypoxia	1	1 (3)	0	1 (1)
Respiratory disorder	1	1 (3)	0	1 (1)

Brain tumors included anaplastic astrocytoma, anaplastic ependymoma glioblastoma, atypical meningioma, high-grade glioma (not otherwise specified), and pleomorphic xantoastrocytoma.

SAE, serious adverse event.

**Table 5:** SAEs in pediatric patients with malignant brain tumors, by diagnosis.

Preferred term, n (%)	Total events (n = 28)	Diagnosis		Total (N = 81)
		Newly diagnosed (n = 41)	Recurrent (n = 40)	
Patients with ≥ 1 SAE	–	3 (7)	8 (20)	11 (14)
Seizure	5	1 (2)	4 (10)	5 (6)
Infection	3	1 (2)	2 (5)	3 (4)
Brain edema	2	1 (2)	1 (3)	2 (2)
Hypertension	2	0	2 (5)	2 (2)
Tachycardia	2	0	2 (5)	2 (2)
Adverse drug reaction	1	1 (2)	0	1 (1)
Colitis	1	0	1 (3)	1 (1)
Nausea/vomiting	1	0	1 (3)	1 (1)
Respiratory tract infection	1	0	1 (3)	1 (1)
Altered state of consciousness	1	0	1 (3)	1 (1)
Cerebral hemorrhage	1	0	1 (3)	1 (1)
Headache	1	0	1 (3)	1 (1)
Hemiparesis	1	0	1 (3)	1 (1)
Hypersomnia	1	0	1 (3)	1 (1)
Paresis	1	0	1 (3)	1 (1)
Speech disorder	1	0	1 (3)	1 (1)
Dyspnea	1	1 (2)	0	1 (1)
Hypoxia	1	0	1 (3)	1 (1)
Respiratory disorder	1	0	1 (3)	1 (1)

Brain tumors included: anaplastic astrocytoma, anaplastic ependymoma glioblastoma, atypical meningioma, high-grade glioma (not otherwise specified), and pleomorphic xantoastrocytoma.

SAE, serious adverse event.

In the total population, 42 (52%) patients experienced ≥1 AE potentially related to TTFields therapy; all were non-serious with skin reaction being the most common (36%) (**Tables 6**, **7**). Other potentially TTFields therapy-related AEs included headache (12%), heat sensation (beneath array warm sensation; warmth [12%]), electric sensation (beneath array tingling sensation; tingling [10%]), fatigue/malaise (6%), medical device pain (4%), and medical device discomfort (2%). Similar incidences of potentially TTFields therapy-related AEs were reported across age groups (**Table 4**).

**Table 6:** Tumor Treating Fields therapy-related AEs in pediatric patients with malignant brain tumors, by age.

Preferred term, n (%)	Total events (n = 99)	Age subgroup		Patients (N = 81)
		Children (<13 years) (n = 40)	Adolescents (13–17 years) (n = 41)	
Patients with ≥1 related AE	–	19 (48)	23 (56)	42 (52)
Skin reaction	48	14 (35)	15 (37)	29 (36)
Electric sensation*	14	3 (8)	5 (12)	8 (10)
Headache	11	2 (5)	8 (20)	10 (12)
Heat sensation^†^	11	3 (8)	7 (17)	10 (12)
Fatigue/malaise	5	3 (8)	2 (5)	5 (6)
Medical device pain	4	2 (5)	1 (2)	3 (4)
Medical device discomfort	2	0	2 (5)	2 (2)
Alopecia	1	0	1 (2)	1 (1)
Medical device site hyperhidrosis	1	1 (3)	0	1 (1)
Medical device site ulcer	1	1 (3)	0	1 (1)
Insomnia		0	1 (2)	1 (1)

Brain tumors included: anaplastic astrocytoma, anaplastic ependymoma glioblastoma, atypical meningioma, high-grade glioma (not otherwise specified), and pleomorphic xantoastrocytoma.

*Beneath array tingling sensation; tingling.

^†^Beneath array warm sensation; warmth.

AE, adverse event.

**Table 7:** Tumor Treating Fields therapy-related AEs in pediatric patients with brain tumors, by diagnosis.

Preferred term, n (%)	Total events(n = 99)	Diagnosis		Patients (N = 81)
		Newly diagnosed (n = 41)	Recurrent (n = 40)	
Patients with ≥ 1 related AE	–	25 (61)	17 (43)	42 (52)
Skin reaction	48	19 (46)	10 (25)	29 (36)
Electric sensation*	14	4 (10)	4 (10)	8 (10)
Headache	11	6 (15)	4 (10)	10 (12)
Heat sensation^†^	11	6 (15)	4 (10)	10 (12)
Fatigue/malaise	5	3 (7)	2 (5)	5 (6)
Medical device pain	4	2 (5)	1 (3)	3 (4)
Medical device discomfort	2	1 (2)	1 (3)	2 (2)
Alopecia	1	1 (2)	0	1 (1)
Insomnia	1	1 (2)	0	1 (1)
Medical device hyperhidrosis	1	1 (2)	0	1 (1)
Medical device ulcer	1	1 (2)	0	1 (1)

Brain tumors included anaplastic astrocytoma, anaplastic ependymoma glioblastoma, atypical meningioma, high-grade glioma (not otherwise specified), and pleomorphic xantoastrocytoma.

*Beneath array tingling sensation; tingling.

^†^Beneath array warm sensation; warmth.

AE, adverse event.

**Figure 2:**
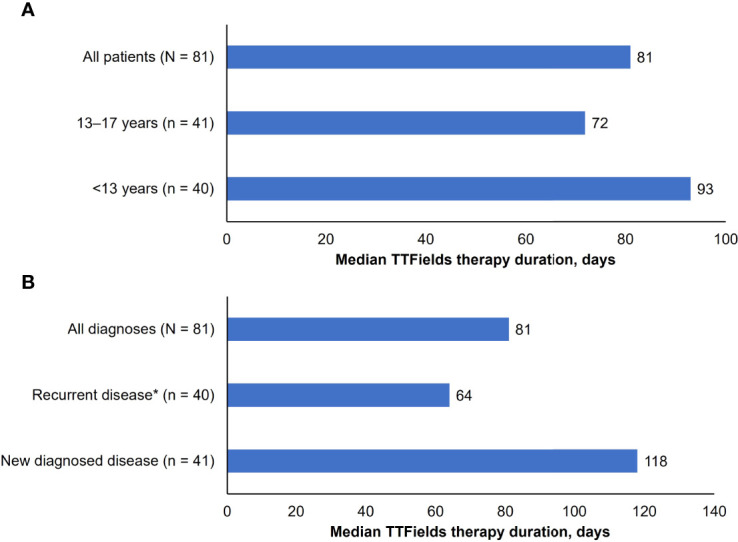
Median TTFields duration of usage (days): **(A)** by age. Brain tumors included: anaplastic astrocytoma, anaplastic ependymoma glioblastoma, atypical meningioma, high grade glioma (not otherwise specified), and pleomorphic xantoastrocytoma. *The group of patients with recurrent disease includes two patients with unknown treatment duration. **(B)** by diagnosis.

The majority of pediatric patients included in this analysis used the second-generation NovoTTF-200A System versus the older NovoTTF-100A System (82% vs. 18%, respectively). Of the patients who used the first-generation NovoTTF-100A device, three (20%) patients switched to the second-generation NovoTTF-200A during treatment. Median treatment duration (range) was 81 days (6–1471 days), and by age group, 93 days (8–956 days) in children, and 72 days (6–1471 days) in adolescents (**Figure 2A**). Treatment duration was longer in newly diagnosed patients (118 days, 8–1471 days) than patients with recurrent disease (64 days, 6–956 days) (**Figure 2B**). Usage data was available for 48 (59%) patients. Overall, median device usage (range) was 75% (4–92), 80% (4–92) for children, 76% (range: 42–92) for adolescents, 79% (7–92) for patients with recurrent disease, and 65% (range: 4–91) for patients with newly diagnosed disease.

## Discussion

This post-marketing surveillance study investigated the safety of TTFields therapy in pediatric patients with malignant brain tumors in a real-world clinical setting. It is important to note that, although the sample size in this study was relatively small, real-world incidences of HGGs and other aggressive CNS tumors are rare in these age groups and therefore this is not unexpected. Baseline characteristics suggested the study population was generally representative of the real world (29). The exception to this was the distribution of patients amongst the age groups; based on epidemiological knowledge, there should have been a greater number of adolescent patients versus child patients (30, 31).

AEs potentially related to TTFields therapy were predominantly dermatologic in nature and generally mild. As with previous studies, no treatment-related systemic or SAEs were reported (18, 19, 23). Median treatment duration was 81 days with a maximum of 1471 days, whilst median usage was 75% – in line with minimum recommended usage. Median treatment duration was longer in children versus adolescents and in those with newly diagnosed versus recurrent disease. As such, age does not appear to be a limiting factor for duration of use.

Results presented here show that TTFields therapy was generally well-tolerated with no new safety signals in this pediatric patient population. The safety profile was in line with data from clinical and post-marketing studies of adult and pediatric patients with GBM, and with those reported in clinical case publications and interim results of the PBTC-048 pediatric study (23–27, 32). The fact that treatment duration and usage was high also supports the tolerability of TTFields therapy.

TTFields therapy-related skin AEs were common, but generally manageable. Skin AEs typically manifest as localized reactions beneath the device arrays and can include contact dermatitis, hyperhidrosis, and xerosis or pruritus. Importantly, these skin reactions can generally be managed with simple prophylactic measures, including maintaining skin health and frequent array change/repositioning, reshaving of the scalp, and appropriate topical treatments such as corticosteroids (33). Furthermore, short treatment breaks may be effective in helping to resolve dermatologic AEs, in addition to topical therapies (33).

Since available therapeutic options for recurrent pediatric brain tumors, particularly HGG, are associated with significant systemic side effects (34), TTFields therapy may be a reasonable and viable treatment option for this patient population, given the lack of systemic side effects. Furthermore, due to its tolerability, TTFields therapy could be added to maintenance chemotherapy or targeted therapy after surgical resection followed by radiochemotherapy for the treatment of patients with newly diagnosed brain tumors. However, data on TTFields therapy and concomitant TMZ (or alternate therapy) and/or radiochemotherapy were not collected for pediatric patients in this post-marketing surveillance safety analysis due to the retrospective nature of the study.

Differences in treatment duration and usage between age groups (children vs. adolescents) could potentially be explained by parent/caregiver influence over younger patients in terms of time spent wearing the device and responsibilities pertaining to decisions to discontinue treatment. Although the treatment duration was higher in newly diagnosed disease, median usage was slightly higher in patients with recurrent disease, perhaps reflecting parents’/caregivers’ motivation and awareness that efficacy improves with increased usage, especially in the context of aggressive recurrent disease (35–37).

An important consideration when using TTFields therapy in pediatric patients is the fact that the field generator and battery must be carried while receiving treatment. The newer NovoTTF-200A System would likely be more practical for pediatric patients due to the smaller size and lighter weight versus the NovoTTF-100A. In one recent case study of a 3-year-old child diagnosed with ndGBM (infratentorial location), the patient’s use of TTFields therapy was initially low, at approximately 40% in month 2 of treatment. However, in months 5 and 6, TTFields therapy use increased to approximately 80% and the average treatment usage was estimated to be 75.87% over months 4–8, which is in line with the minimum recommended usage of at least 18 h/day (12, 19, 32, 35). A radiological response was recorded 1 year after the patient’s initial diagnosis and no TTFields therapy-related AEs were reported. The fact that usage of TTFields therapy increased over time, even though the patient was very young, is promising. Additionally, this patient had an infratentorial tumor and the array layout had not been assessed clinically (as TTFields therapy is only approved for supratentorial tumors), so a computer simulation-based study was used to demonstrate feasibility and guide array placement. This case study helps illustrate that TTFields therapy is feasible in very young pediatric patients, although it should be noted that this assumption is based on data from one case study and should be rigorously tested in a clinical trial setting.

The potential for a subjective impact on QoL should also be considered in pediatric patients. Interim health-related QoL data from the PBTC-048 trial showed that TTFields therapy had no significant impact on QoL, except that the patients felt more self-conscious after treatment (i.e. more exposed with a shaved head) than before (38). Results from the phase 3 EF-14 clinical study showed that TTFields therapy did not negatively affect role, social, and physical function in adult patients when added to maintenance TMZ (20). Deterioration-free survival was also significantly longer with TTFields therapy for global health and physical and emotional functioning relative to TMZ alone. Furthermore, a recent real-world analysis showed that changes to HRQoL were maintained during TTFields therapy use (20).

Given the impact on QoL that pediatric patients Experience due to their diagnosis. e.g. disruption to schooling, it is important to monitor and manage events that could affect their QoL, including an assessment of health-related QoL measures prior to the initiation of TTFields therapy. However, there are limited tools available to assess the impact of AEs and physical changes on children’s QoL (39). New tools and more studies are urgently needed to better identify and monitor the effects of any treatment on QoL (38).

Significantly, no new safety signals were observed in this post-marketing safety surveillance analysis, which is an important observation considering that a child’s brain is still in development. Although *in vivo* and *in vitro* data have shown that TTFields selectively targets cancer cells, with minimal impact on healthy cells, additional data showing the effects of TTFields on myelinating cells would further support the safety of TTFields therapy in pediatric patients (40–42).

This study has several limitations. Due to the retrospective nature of the study, the analysis was descriptive with no formal statistical comparisons. AE data were not actively solicited, and therefore some patient information (e.g. concomitant treatment, full clinical history, and reasons for treatment discontinuation) was unavailable or only available for a limited number of patients. Omission of this information could have an impact on how the results of this study were interpreted. For example, certain medications such as steroids or chemotherapy can increase skin fragility and may have predisposed patients to skin AEs (43, 44), contributing to the occurrence of skin AEs under the arrays. Lastly, as this analysis focused on safety, there are no efficacy data available.

Due to the design and limitations of this study, no definitive statements can be made, and further investigation is needed given the significant unmet medical need for tolerable and effective treatment options in the heavily burdened pediatric population with brain tumors (34, 45). Currently, three clinical trials evaluating the feasibility and safety of TTFields therapy in pediatric patients with brain tumors are ongoing: the HUMC 1612 trial (NCT03128047) of TTFields therapy with concomitant temozolomide and bevacizumab in children with recurrent HGG and ependymoma, the PBTC-048 trial (NCT03033992) of TTFields monotherapy in children with recurrent or progressive supratentorial HGG and ependymoma, and a study in Japan assessing TTFields therapy in children (aged 5–17 years old) with ndGBM or rGBM (jRCTs032200423) (11, 46). PBTC-048 has opened a second stratum for children with DIPG and concomitant radiotherapy and maintenance TTFields therapy. Results from these studies will provide much-needed clinical efficacy and safety data on TTFields therapy in pediatric patients.

## Conclusion

Data presented here suggest a favorable safety profile for TTFields therapy, with predominantly mild to moderate localized skin AEs and no unexpected toxicities in pediatric patients with HGGs and other malignant brain tumors. These results are aligned with safety data from previous reports in adult patients as well as pediatric and adolescent patients (18, 19, 23–27, 37). These data along with previous reports suggest broad safety of TTFields therapy across populations and age groups and that TTFields therapy is a feasible treatment option for pediatric patients with brain tumors, including HGGs. Their utility requires additional investigation in this patient population.

## Data availability statement

The datasets generated during and/or analyzed during the current study are available for 3-years after date-of-publication. Please contact Sharon Perez, Vice President, Global Medical Device Safety, (SPerez@novocure.com) for inquiries about access.

## Ethics statement

Ethical review and approval was not required for the study on human participants in accordance with the local legislation and institutional requirements. Written informed consent from the participants’ legal guardian/next of kin was not required to participate in this study in accordance with the national legislation and the institutional requirements.

## Author contributions

All authors contributed to the conception and design of the study, data analysis and interpretation. All authors contributed to the article and approved the submitted version.

## Funding

Novocure Ltd was responsible for data collection and analysis. All fees related to publication were funded by Novocure Ltd.

## Acknowledgments

The authors would like to thank the patients, their families and all investigators involved in this study. Statistical support was provided by Julia Stindl, PhD, of Global Medical Affairs, Novocure Inc., Medical writing support under the guidance of the authors was provided by Imogen Francis, BSc, and Melissa Purves PhD, of Prime, Knutsford, UK. Review of the manuscript was provided by Adrian Kinzel, MD, Leonardo Lustgarten, MD, Huda Ismail Abdullah, PhD, and Chelsea Higgins, PhD, CMPP, of Global Medical Affairs, Novocure Inc., Writing and editorial support provided by Prime was funded by Novocure Inc. and conducted according to Good Publication Practice guidelines (Link). The Sponsor was involved in the study design, collection, analysis, and interpretation of data, as well as data checking of information provided in the manuscript. However, ultimate responsibility for opinions, conclusions, and data interpretation lies with the authors.

## Conflict of interest

The authors declare that this study received funding from Novocure Ltd. The funder had the following involvement in the study: data collection and analysis. Editorial assistance and all costs related to publication were funded by Novocure Ltd. SG and SK declare funding for unrelated clinical research, from Novocure Ltd.

## Publisher’s note

All claims expressed in this article are solely those of the authors and do not necessarily represent those of their affiliated organizations, or those of the publisher, the editors and the reviewers. Any product that may be evaluated in this article, or claim that may be made by its manufacturer, is not guaranteed or endorsed by the publisher.
